# CEACAM6’s Role as a Chemoresistance and Prognostic Biomarker for Pancreatic Cancer: A Comparison of CEACAM6’s Diagnostic and Prognostic Capabilities with Those of CA19-9 and CEA

**DOI:** 10.3390/life11060542

**Published:** 2021-06-09

**Authors:** Benediktas Kurlinkus, Marija Ger, Algirdas Kaupinis, Eugenijus Jasiunas, Mindaugas Valius, Audrius Sileikis

**Affiliations:** 1 Clinic of Gastroenterology, Nephrourology and Surgery, Institute of Clinical Medicine, Faculty of Medicine, Vilnius University, LT-03101 Vilnius, Lithuania; audrius.sileikis@mf.vu.lt; 2 Proteomics Center, Institute of Biochemistry, Vilnius University Life Sciences Center, LT-10257 Vilnius, Lithuania; marija.ger@bchi.vu.lt (M.G.); algirdas.kaupinis@gf.vu.lt (A.K.); mindaugas.valius@bchi.vu.lt (M.V.); 3 Centre of Informatics and Development, Vilnius University Hospital Santaros Klinikos, LT-08661 Vilnius, Lithuania; eugenijus.jasiunas@santa.lt

**Keywords:** pancreatic cancer, CEACAM6, prognostic biomarker, chemoresistance

## Abstract

Survival rates from pancreatic cancer have remained stagnant for decades due to the heterogenic nature of the disease. This study aimed to find a new advanced biomarker and evaluate its clinical capabilities, thus enabling more individualised pancreatic cancer management. Between 2013 and 2020, 267 patients were included in the study. Surgically collected pancreatic tissue samples were analysed via high-definition mass spectrometry. Carcinoembryonic antigen-related cell adhesion molecule 6 (CEACAM6) was discovered as a possible promising pancreatic cancer biomarker. The predominance of CEACAM6 to pancreatic cancer was validated using antibodies in tissue samples. CEACAM6, carbohydrate antigen 19-9 (CA19-9), and carcinoembryonic antigen (CEA) blood serum concentrations were evaluated for clinical evaluation and comparison. Kaplan–Meier survival analyses were used to evaluate disease-free survival (DFS) and overall survival (OS). Poorer overall survival was significantly dependent on increased CEACAM6 blood serum concentrations (17.0 vs. 12.6 months, *p* = 0.017) in pancreatic cancer patients after radical treatment and adjuvant chemotherapy. Increased CEA and CA19-9 concentrations showed no significant dependencies with survival. Thus, CEACAM6 is a promising new biomarker with significant prognostic value and prediction of chemoresistance properties, enabling the improvement of individualised approaches to patients with pancreatic cancer.

## 1. Introduction

Pancreatic ductal adenocarcinoma (PDAC), the most common type of pancreatic cancer, is emerging as one of the most significant oncological diseases in the European Union (EU). Recent estimations have concluded that pancreatic cancer mortality rates overtook the number of deaths from breast cancer in the EU in 2017. Pancreatic cancer is now the EU’s third-leading cause of cancer-related death, just below lung and colorectal cancers [1]. The only option for curative treatment of this disease is radical surgery, while properly selected chemotherapy or immunotherapy can improve survival. Most cases of pancreatic cancer are identified in the late stages, thus making curative treatment impossible and leading to a poor prognosis. Life expectancy after diagnosis is just 4 to 6 months and has not improved much over the last four decades [2]. This is mostly due to the lack of ways to diagnose the disease at the early stages. Radiological methods are limited in terms of availability, cost, and diagnostic sensitivity. Scientists agree that the first invasive cancer cells are present in the pancreas for several years before they expand across the pancreas or further metastasize [3]. Due to altered metabolism, these cells distinguish themselves by producing and secreting various unique metabolites, including amino acids and proteins. These molecules can be used as biomarkers, providing exceptional potential for early cancer detection [4,5,6]. Another important reason for poor prognosis after pancreatic cancer diagnosis is the heterogenic nature of the disease [7,8,9]. This makes it difficult for clinicians to select the best treatment strategy for each individual patient. A significant prognostic biomarker, especially with the possibility to predict chemoresistance, would enable more individualised patient evaluations and eventually lead to a more balanced treatment plan for each particular patient. A recently published United European Gastroenterology report on pancreatic cancer called for efforts at the highest level to increase research into molecular markers [10].

Carcinoembryonic antigen-related cell adhesion molecule 6 (CEACAM6) belongs to the carcinoembryonic antigen-related cell adhesion molecules (CEACAMs) family. This is a group of proteins found only in mammals [11]. This family belongs to the immunoglobulin superfamily of cell adhesion molecules, which is comprised of a large group of cell surface glycoproteins [12]. Inside the CEACAMs family, there are more than 17 structurally similar members expressed on the apical surfaces of various cells, including epithelial, endothelial and hematopoietic cells [13]. These proteins specialize in cell–cell adhesion and recognition, as well as modulating various cellular processes, including regulation of the cell cycle, tumour suppression, and angiogenesis [11,12,14,15]. Because CEACAMs are crucial for such important processes of the cell cycle, they influence the progression of various cancers, including pancreatic cancer. One of the CEACAMs members, CEACAM6, is particularly promising. Research analysis has already shown its potential to be used as a biomarker, even in pancreatic cancer precursor lesions—pancreatic intraepithelial neoplasia [13,16,17,18]. Since increased CEACAM6 expression is associated with a more aggressive subtype of PDAC, it was identified as a prognostic biomarker, also relevant to evaluating the patients’ response to chemotherapy [13,16,19,20]. However, CEACAM6 analyses performed so far have rarely examined blood serum samples, nor have there been comparisons with biomarkers already in use. Thus, its evaluation for possible clinical use remains incomplete.

In our study, we searched for a new improved biomarker for PDAC and identified and validated CEACAM6 expression predominantly in pancreatic cancer tissue samples. Furthermore, we evaluated CEACAM6 and the conventional biomarkers carcinoembryonic antigen (CEA) and carbohydrate antigen 19-9 (CA19-9) concentrations in blood serum samples from patients with PDAC, chronic pancreatitis (CP), and healthy controls (HC). Finally, we determined optimal concentration cut-off values and evaluated and compared the diagnostic and prognostic capabilities of these three biomarkers.

## 2. Materials and Methods

### 2.1. Study Design, Patients’ Selection and Samples Collection

**Figure 1:**
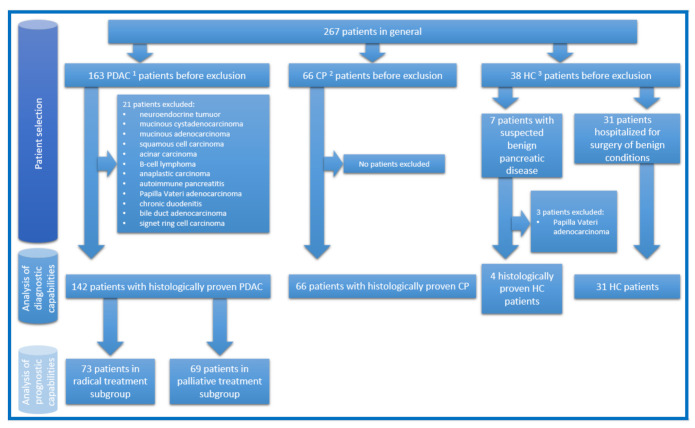
The study design, numbers of patients in each analysed group, and reasons for exclusion. ^1^ Pancreatic ductal adenocarcinoma; ^2^ chronic pancreatitis; ^3^ healthy controls.

This research was approved by Vilnius Regional Ethics Committee for Biomedical Research on 10 September 2013 (approval number 158200-13-675-214). Informed consent was obtained from all subjects involved in the study. Between 20 November 2013 and 6 October 2020, 267 Caucasian patients were included in the study. They were treated in the Vilnius University Hospital Santaros Clinics. Patients were divided into three groups: patients with histologically proven PDAC, patients with histologically proven CP, and patients as HC (patients with histologically proven benign pancreatic disease (pancreatic mucinous or serous cystadenomas), or patients hospitalized for surgery of benign conditions (i.e., inguinal hernia, haemorrhoids). Surgical pancreatic tissue samples and blood serum samples were collected. Patients with PDAC were hospitalized either for radical treatment and underwent pancreaticoduodenectomy/hemipancreatectomy or for palliative treatment and underwent bypass operations and/or biopsies. Patients with CP underwent Frey’s procedure. HC patients underwent standard treatment for their benign conditions or pancreaticoduodenectomy/hemipancreatectomy in cases of benign pancreatic disease. In latter cases, samples are considered as HC as they were taken from adjacent healthy pancreatic tissue. The study design, numbers of patients in each analysed group, and reasons for exclusion are demonstrated in Figure 1. All tissue samples were collected during surgery and flash-frozen in liquid nitrogen for 10–15 min, then further stored at −80 °C until lysate preparation. All blood serum samples were collected directly before surgery and prepared for further storage at −80 °C according to a standardized protocol [21]. None of the patients in the PDAC group received neoadjuvant chemotherapy. Most of the patients with PDAC received adjuvant chemotherapy. All patients with PDAC had subsequent follow up between 0.5 and 5 years.

### 2.2. Proteomic Analysis of Tissue Samples

To examine disease-associated changes in the proteome, high-throughput differential label-free quantitative proteomic analysis of PDAC, CP, and HC patients’ tissue samples was performed using high-definition mass spectrometry (HDMS) technology. In addition, PDAC samples were divided into 2 groups according to diameter: smaller (<2 cm) tumours and larger (>3 cm) tumours. Homogenized samples were lysed using urea/thiourea lysis buffer and sonicated for 1 min at the amplitude of 20% and 0.4 s pulsations of on/off cycles. Lysates were centrifuged at 20,000× *g* for 15 min at 4 °C, and the supernatants were collected and stored at –80 °C. Trypsin digestion was performed according to a modified filter-aided sample preparation protocol, as described previously [22].

Liquid chromatography separation of peptides was performed with the nanoAcquity ultra-performance liquid chromatography system (Waters Corporation, Elstree, UK) on a reversed-phase trap column, as described previously [22]. Data were acquired using MassLynx version 4.1 software (Waters Corporation, Milford, MA, USA) in positive ion mode. Liquid chromatography–mass spectrometry data were collected using data-independent acquisition mode MSE in combination with online ion mobility separation. The trap collision energy of the mass spectrometer was ramped up from 18 to 40 eV for high-energy scans in MSE mode. The trap and transfer collision energy for high-energy scans in HDMS mode was ramped up from 4 to 5 eV and from 27 to 50 eV. The mass range was set to 50–2000 Da with a scan time set to 0.9 s. The reference compound [Glu1]-fibrinopeptide B (Merck, Kenilworth, NJ, USA) was infused continuously (500 fmol/μL at a flow rate 500 nL per minute) and scanned every 30 s for online mass spectrometer calibration purposes. The samples were run in triplicate.

Raw proteomic data files were processed and searched using ProteinLynx Global SERVER (PLGS) version 2.5.3 (Waters Corporation, Milford, MA, USA). The following parameters were used to generate peak lists: (i) the minimum intensity for precursors was set to 150 counts; (ii) the minimum intensity for fragment ions was set to 50 counts; (iii) the intensity was set to 500 counts. Processed data were analysed using trypsin as the cleavage protease. One missed cleavage was allowed, fixed modification was set to “carbamidomethylation of cysteines”, and variable modification was set to “oxidation of methionine”. Minimum identification criteria included 1 fragment ion per peptide, 3 fragment ions per protein, and a minimum of 2 peptides per protein. The false discovery rate (FDR) for peptide and protein identification was determined based on the search of a reversed database generated automatically when the global false discovery rate was set to 4%. The UniProtKB/SwissProt human database (5 February 2018) was used for protein identification.

### 2.3. Tissue Lysates Preparation and Western Blot

Validation of CEACAM6 expression in PDAC tissue samples was performed using the Western blot technique. Eight PDAC samples were used. As a negative control, 8 corresponding pancreatic tissue samples were used. Of them, 4 were CP and 4 were HC. From each tissue sample, a lysate was prepared. Pieces of tissue weighing 0.02 g were ground into smaller pieces and mixed with 240 µL buffer containing TRIS/HCl pH 7.6 and sodium dodecyl sulphate. The solutions were then heated at 100 °C for 5 min. After heating, each sample was disintegrated for 1 min using ultrasound SONOPULS (BANDELIN, Germany) cycle 4 × 10% power 20%. The cycles of heating and ultrasound disintegration were repeated one more time before final centrifugation at G at a temperature of 22 °C for 15 min. The transparent solutions were isolated from the remaining debris for further processing. From each of them, 210 µL of the solution was taken, DTT was added to 0.1 M concentration, and the samples were stored at −20 °C. The remainder left after centrifugation was used for the measurement of total protein concentrations using the BCA Protein Assay Kit (Thermo Fisher Scientific, Waltham, MA, USA) according to the manufacturer’s instructions and a BioPhotometer Spectrophotometer (Eppendorf, Hamburg, Germany). Using this information, all stored solutions were diluted to the same protein concentration of 50 µg/µL.

For Western blot analysis, tissue lysates were resolved using 10% SDS-PAGE, and 30 µL of lysate were loaded into each track. PageRuler™ Prestained Protein Ladder, 10 to 180 kDa (Thermo Fisher Scientific, Waltham, MA, USA) was used as a marker. The proteins were transferred to polyvinylidene difluoride membrane (Bio-Rad, Hercules, CA, USA) and blocked in Blotto (0.9% NaCl, 8 mM Tris HCl, 2 mM Tris, 1% skimmed milk, 0.025% Tween-20, 0.05% NaN3) solution. Staining was done using primary antibodies: CEACAM6 mouse monoclonal antibody, clone 9A6, cat. Nr. sc-59899 (Santa Cruz Biotechnology Inc., Dallas, TX, USA) and β-actin mouse monoclonal antibody, clone # 937215, cat. Nr. MAB8929 (Bio-Techne, Minneapolis, MN, United States). As secondary antibodies, IRDye^®^ 800CW Goat anti-Mouse IgG (LI-COR Biosciences, Lincoln, NE, USA) were used with an effect time of 30 min. For scanning, the membrane scanner LI-COR Odyssey 9120 was used together with its software package, Odyssey (LI-COR Biosciences, Lincoln, NE, USA).

### 2.4. Enzyme Linked Immunosorbent Assay (ELISA) and Chemiluminescent Microparticle Immunoassay (CMIA)

For quantitative analysis of CEACAM6, CEA, and CA19-9 in peripheral blood serum samples, two techniques were used. ELISA was used for the quantification of CEACAM6 and CMIA for CEA and CA19-9. Blood samples of 142 patients with PDAC, 66 patients with CP, and 31 patients as HC were analysed. For the quantitative analysis of CEACAM6 in the blood serum, CEACAM6 ELISA kits (catalog number: MBS7203989 (MyBiosource, San Diego, CA, USA)) were used. The manufacturer’s recommendations were used for the preparation of samples and reagents, the assay procedure, and the calculation of the results. CEA and CA19-9 concentrations in blood serum samples were determined using the CMIA technique. The ARCHITECT iSystem with reagents ARCHITECT CA 19-9XR assay (Abbott, Chicago, IL, USA) and ARCHITECT CEA assay (Abbott, Chicago, IL, USA) were used. The manufacturer’s recommendations were used for sample and reagent preparation, assay procedures, and the calculation of results.

### 2.5. Statistics

Statistical analysis was performed using software: R statistical software package V 4.0.2 (© The R Foundation for Statistical Computing, https://www.r-project.org/foundation/, accessed on 9 June 2021), Rstudio Version 1.3.959 © 2009–2020 RStudio, Inc., IBM SPSS Statistics V.23, G*Power V. 3.1.9.4 Universität Düsseldorf, Germany. Shapiro-Wilk and Kolmogorov-Smirnov (K-S) tests were used to check the data for normality. Interval variables, which were not normally distributed, were described by medians and interquartile ranges (IQR). The nominal variables were characterized by their frequencies and percentage across the corresponding subset of the sample. For testing relationships between categorical variables, the chi-square (χ^2^) tests of independence were used. When the frequencies of the values were below 5, for testing relationships between categorical variables, we used Fisher’s exact test. To estimate a statistically significant relationship between groups of variables, we used the Kruskal-Wallis rank-sum test. The strength of the relationship was measured by the eta squared based on the H-statistic (eta^2^ [H]) effect size. When eta^2^ [H] = 0.01 ≤ 0.06, we had a small effect; when eta^2^ [H] = 0.06 ≤ 0.14, we had a moderate effect; and when eta^2^ [H] ≥ 0.14, we had a large effect. The Youden index was used to calculate optimal blood serum concentration cut-off values for each biomarker according to its prognostic capabilities. Kaplan-Meier analysis (log-rank test) was used for disease-free survival (DFS) and overall survival (OS) analysis excluding 90-day mortality (15 events) and non-cancer-related deaths (5 events). The relationships between variables were evaluated as statistically significant with a *p*-value less than 0.05 (*p* < 0.05) and a statistical test power equal to 0.95 (1 − ß = 0.95).

## 3. Results

### 3.1. Proteomic Identification of CEACAM6 as Potential PDAC Biomarker

To identify potential PDAC biomarkers, we undertook an in-depth proteomic analysis of 19 tissue specimens surgically derived from healthy individuals (HC; 4 specimens), patients with CP (5 specimens), and patients with PDAC (10 specimens). In addition, specimens from PDAC patients were sorted into two groups: five specimens from smaller (<2 cm) tumours and five specimens from larger (>3 cm) tumours, aiming to identify biomarkers specific to the earlier or later stages of the disease. A total of 3627 proteins in all patient proteomes were identified and quantified, and 350 proteins were significantly increased in tumour proteomes (*p* value < 0.05; fold change > 1.5) (Supplementary Materials: Table S1). Ten proteins were unique for all PDAC, but not for HC or CP samples. Of them, only CEACAM6 has the possibility to be secreted into the blood serum, thus having the potential for clinical application. We further investigated CEACAM6 because it is considered as a promising new PDAC biomarker by other researchers [13,16,23].

### 3.2. CEACAM6 Expression in PDAC Tissue Specimens

**Figure 2:**
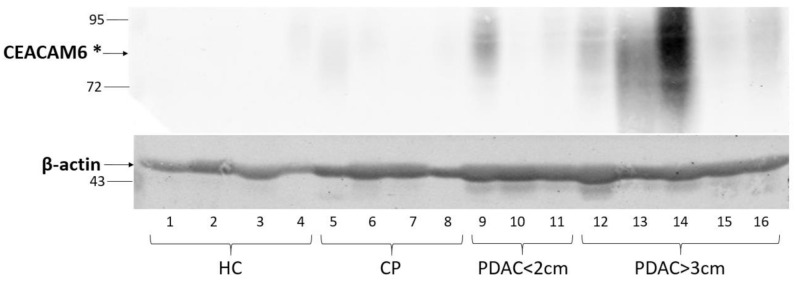
Western blot analysis of CEACAM6 expression in pancreatic HC, CP, and PDAC tissue lysates. β-actin was used as a loading control. * Carcinoembryonic antigen-related cell adhesion molecule 6.

To validate the specificity of CEACAM6 to PDAC but not HC or CP tissue samples, we assayed the expression of CEACAM6 with specific antibodies. Western blot analysis and densitometry readings revealed that CEACAM6 is expressed in PDAC tissue samples with a tendency for increasing expression as the tumour diameter increases. Additionally, no detectable expression of CEACAM6 was observed in HC tissue samples and only a trace of CEACAM6 expression was found in CP tissue samples (Figure 2, Table 1). These results confirm the predominant CEACAM6 expression in PDAC tissues.

### 3.3. Evaluation of CEACAM6, CEA and CA19-9 Concentrations in Patients’ Blood Serum, Diagnostic Capability of CEACAM6

According to a previous study, CEACAM6 is secreted into and can be detected in the blood serum [13]. To evaluate the ability of CEACAM6 to separate PDAC patients from those with CP and HC, we analysed CEACAM6 blood serum concentrations from 239 patients with ELISA. Before the analysis, patients were divided into three groups: patients with PDAC, patients with CP, and patients as HC. The demographics of the groups were analysed to evaluate general parameters and the overall health condition of each group (Table 2). Analysis revealed significant differences between the groups in all evaluated demographical parameters. These changes might have influenced further results, but they cannot be avoided due to the nature of each group.

To compare the diagnostic capability of CEACAM6 with other biomarkers, we further analysed concentrations of already known PDAC biomarkers (CEA and CA19-9) in the same blood serum samples with CMIA. Differences in the median values of analysed biomarker concentrations between the three groups reached statistical significance and are shown in Table 3. The analysis confirmed that CEA and CA19-9 have diagnostic potential, as their concentrations were significantly increased in the PDAC group. The concentration of CEACAM6 was significantly increased in one of the control groups, CP patients, thus restricting its diagnostic potential.

### 3.4. Comparative Analysis of CEACAM6 as a Prognostic Biomarker

Finally, we investigated the prognostic potential of CEACAM6 and compared it to the prognostic capabilities of CEA and CA19-9. Aiming to evaluate the analysed biomarker associations to various oncological and demographical parameters, we evaluated the dependency of CEACAM6, CEA, and CA19-9 blood serum concentrations from these parameters in PDAC patients. The results of this analysis are represented in Table 4. Our study revealed that CEACAM6 and CEA blood serum concentrations are less dependent on the evaluated oncological and demographical parameters compared to CA19-9 concentration. This means CEACAM6 could be considered as a partially independent candidate biomarker for PDAC.

**Figure 3:**
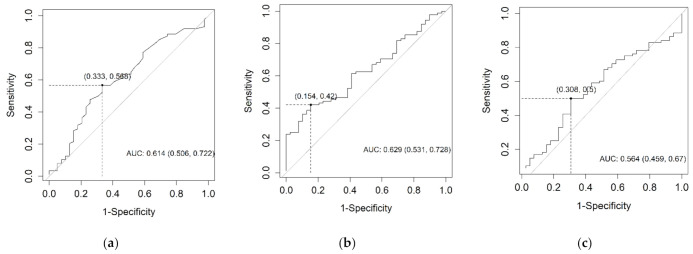
The ROC curves for CEA (**a**), CEACAM6 (**b**), and CA19-9 (**c**) according to their prognostic capabilities.

For the survival analysis, optimal cut-off values for each biomarker blood serum concentration needed to be determined. Since diagnostic CEACAM6 potential was rejected, cut-off values were calculated according to the prognostic capabilities of the biomarkers using the Youden index. The determined cut-off values were CEA 2.6 mkg/L; CEACAM6 3.018 ng/mL; CA19-9 308.85 kU/L. ROC curves for each biomarker are represented in Figure 3. The established cut-off values of all three biomarkers were applied in the subsequent survival analysis.

PDAC patients in our study underwent different types of surgical treatments, which may have had an impact on their survival. For this reason, before survival analysis, we divided the PDAC patient group into two subgroups depending on the type of treatment patients received—radical or palliative. A total of 73 patients in the radical treatment subgroup underwent pancreaticoduodenectomy or hemipancreatectomy, while 69 patients in the palliative treatment subgroup underwent bypass operations and/or biopsies. The latter subgroup included patients with resectable stages of PDAC who refused surgery or could not undergo radical surgery due to comorbidities. The characteristics of each subgroup are represented in Table 5.

**Figure 4:**
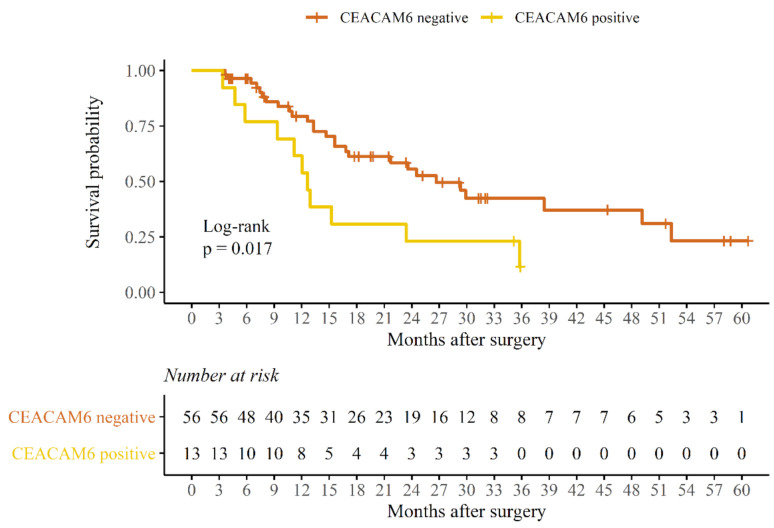
Kaplan-Meier curve representing the dependency of CEACAM6 blood serum concentration values on overall survival in PDAC patients after radical treatment.

To evaluate the prognostic potential of CEACAM6 and to compare it with CEA and CA19-9, a Kaplan-Meier survival analysis was performed. This analysed the impact of these biomarker blood serum concentrations above the cut-off value on survival. Patients with 90-day mortality and non-cancer-related deaths were excluded to evaluate only cancer-related mortality. The median OS and median DFS were calculated for each biomarker in both PDAC subgroups (Table 6). No difference in DFS or OS for either biomarker was detected in the palliative treatment subgroup. However, in the radical treatment subgroup, only patients with CEACAM6 blood serum concentration above the cut-off value were characterized by statistically significant poorer median OS (12.6 months OS for CEACAM6-positive vs. 17.0 months OS for CEACAM6-negative, *p* = 0.017) (Figure 4). No statistically significant dependencies on DFS or OS from either CEA or CA19-9 biomarkers were obtained in the latter subgroup. Because OS, but not DFS, was dependent on CEACAM6 blood serum expression, this might indicate that CEACAM6 cannot predict the timing of disease relapse. However, as it occurs, the disease tends to become more aggressive. Since most of the radical treatment subgroup patients received adjuvant chemotherapy (Table 5), it can be concluded that the level of CEACAM6 in the blood serum has chemoresistance prediction properties in PDAC patients after radical treatment.

## 4. Discussion

The reason why PDAC has emerged as an increasingly significant healthcare issue might be due to the lack of relevant scientific research in the field. Despite the fact that mortality is rising and survival rates have remained unchanged for decades, pancreatic cancer still receives less than 2% of all cancer research funding in Europe [10,24]. From all treatment modalities available, only surgery offers the possibility of a cure if accessed at early stages. Early identification of this disease remains a major issue. Another important issue is the heterogeneity of PDAC, which is due to the nature of its oncogenesis [7,8,9]. This makes it difficult for clinicians to predict further disease development and prognosis, and thus the decision for the best treatment option for each individual patient remains debatable. A new biomarker could shed more light onto these issues; the current biomarkers for PDAC (CA19-9 and CEA) are limited in diagnostic and prognostic capabilities.

In our study, we aimed to find a new advanced PDAC biomarker. After HDMS revealed candidate biomarkers, we determined that CEACAM6 has the highest potential to become a novel biomarker for PDAC. After its validation solely in PDAC tissue, we further analysed blood serum samples for its expression. To evaluate the clinical significance of CEACAM6, we also measured blood serum concentrations of other biomarkers already in use for PDAC (CEA and CA19-9). Although its diagnostic capabilities were insufficient, survival analysis revealed that it has significant prognostic potential and can help to predict chemoresistance. Similar properties of CEACAM6 were investigated and described by other scientists.

Various mechanisms of how CEACAM6 affects pancreatic cancer progression were already identified. Duxbury et al. in 2004 announced a number of publications regarding CEACAM6 and its role in PDAC development. This team noted that CEACAM6 gene silencing reduces the metastatic ability of pancreatic adenocarcinoma cells by impairing anoikis resistance in PDAC cell lines and tested it with a nude mouse orthotopic xenograft model [25]. Another research group led by Duxbury determined that the antibody-mediated cross-linking of CEACAM6 induced a significant increase in cellular resistance to anoikis [26]. Anoikis is a subset of apoptosis normally induced by inadequate cell-substrate adhesion, while resistance to anoikis is a feature of malignancy and determines metastatic potential and tumorigenesis. It was proved that CEACAM6 overexpression has an effect on cellular invasiveness towards insulin-like growth factor I, which has a critical role in the malignant behaviour of pancreatic cancer cells [27]. CEACAM6 was identified to promote PDAC cell interactions with the extracellular matrix via its cross-talk with alphavbeta3 integrin, thus contributing to the invasive and metastatic potential of the cells [28]. Another published study investigated two PDAC cell lines (Capan2 and BxPC3) with modified CEACAM6 expression and their effect on subcutaneously xenografted mice. It was concluded that CEACAM6 expression can modulate the invasive PDAC phenotype through alterations in cellular MMP-9 activity [29]. Gebauer et al. [13] also performed in vivo experiments with mice inoculated with CEACAM knock-down cells. Experimental animals showed a prolonged overall survival in comparison to the control group. On the other hand, the same group showed an increased incidence of pulmonary metastasis. Epithelial–mesenchymal transition is a process which involves the loss of cell–cell adhesions and in PDAC leads to the gain of invasive and metastatic capabilities together with chemoresistance. Chen et al. [20] suggested that CEACAM6 promotes PDAC spread by means of this process via the ZEB1/ZEB2 pathway. CEACAM6 modulation of PDAC cell proliferation via the expression of cyclin D1/CDK4 was also determined by Yan et al. [30].

It is very likely that these oncogenic mechanisms associated with CEACAM6 start to take effect very early, since this protein is confirmed to be expressed even at the preneoplastic state of PDAC, pancreatic intraepithelial neoplasia [13,16,17,18]. Some of these preneosplastic changes are present in patients with CP [17,31,32,33]. This is one of the reasons why CP is considered as a precancerous state by some specialists. CEACAM6 expression was already reported in CP tissue samples in several publications, but the number of analysed samples was low [17,34]. It was also identified in CP patients’ bile and blood serum [13,35]. These findings are similar to our determination that CEACAM6 blood serum concentrations were higher in CP patients than in PDAC patients (Table 3). The diagnostic value of CEACAM6 could be considered to be poor because differentiation between these two conditions is very important in clinical work. In fact, our results may support the results of Sharma et al., who found that the expression of CEACAM6 in the tissue samples of the precancerous state of esophageal cancer was higher than in the actual esophageal cancer [36]. On the other hand, CEACAM6 could has an additional role as a biomarker for the management of CP patients. To this day, no research regarding such clinical utility of CEACAM6 has been done.

Since CEACAM6 possesses significant features for pancreatic cancer development, it was investigated as a potential treatment target. Cheng et al. determined that antibodies targeting CEACAM6 can reduce PDAC cell line angiogenesis, invasion, and MMP-9 activity, three properties important for tumour growth and metastasis [23]. The same research also compared their effect with standard chemotherapy and proved that these antibodies are superior to gemcitabine in terms of their ability to reduce angiogenesis and MMP-9 activity. CEACAM6 demonstrated the potential to be used as a therapeutic target for PDAC using an antibody-drug conjugate-based therapy approach [18]. In vitro analysis made by Duxbury et al. [19] determined that the expression of CEACAM6 is associated with the gemcitabine chemoresistance of PDAC cells, as in our results, where patients with higher CEACAM6 blood serum concentrations did not have a significant therapeutic impact from adjuvant chemotherapy, as shown by poorer OS (Table 6, Figure 4). These findings could mean that CEACAM6 may help clinicians to identify a group of patients with chemoresistant but targeted therapy- and/or immunotherapy-responsive PDAC. One ongoing clinical trial is analysing the effect of CAR2Anti-CEA CAR-T cell infusion to the hepatic artery for patients with pancreatic cancer and CEA+ liver metastases that are resistant to standard therapy [37]. The results of this trial will hopefully shed even more light on this possible clinical role of CEACAM6.

Our analysis revealed that CEACAM6 and CEA blood serum concentrations are less dependent on various oncological and demographic parameters compared to CA19-9 (Table 4). CEA and CEACAM are almost identical in their association to parameters reported, with the only difference being the stage. Given that they are both associated with T, M, perineural invasion, and perivascular invasion, the difference in stage could be linked to sample size. The more dependent CA19-9 significantly correlated with the same parameters as CEACAM6 and CEA, but also with the parameters N and tumour diameter. A similar analysis conducted by Gebauer et al. concluded that CEACAM6 blood serum concentrations significantly correlate with the parameters G and M, while CEA did not correlate with any of analysed parameters [13]. However, the latter study analysed fewer parameters, and patient groups were smaller.

In our research, we compared patients’ demographical parameters between different groups: PDAC, CP and HC. We concluded that there were significant differences between the groups in all evaluated parameters (Table 2). This might have had an effect on biomarker expression, but we could not adjust the study accordingly due to the limited number of patients available. We understand that other studies with a similar study design might run into the same issue, since each patient group has a tendency to experience certain changes in their health condition or demographical parameters (e.g., median BMI, median age, gender ratio, incidence of diabetes mellitus). While analysing CEACAM6 blood serum expression with a similar study structure, Gebauer et al. reported the gender ratio only for PDAC and HC patients, but for CP patients, these data are missing [13]. In the same study, the median age was reported only for PDAC and CP patients, but for HC patients, information about age is also missing.

CEACAM6 was reported to be overexpressed not only in the cancer tissue and blood serum of PDAC patients, but also in their bile. Farina et al. [35] concluded that CEACAM6’s concentration is conspicuously elevated in bile samples from patients with PDAC and cholangiocarcinoma, but not in those from chronic pancreatitis or gallstone-induced stenosis. This makes it a possible biomarker for differentiation between benign and malignant strictures, a subject that still remains a major clinical issue.

Although a significant number of studies have been conducted already, reporting a link between higher CEACAM6 concentration and the more aggressive type of PDAC, few trials have investigated its prognostic capabilities. Two trials available on this subject have analysed immunohistochemically stained tissue microarray specimens [13,16]. In these studies, positive CEACAM6 expression was associated with unfavourable OS and DFS. One additional trial analysed The Cancer Genome Atlas data from PDAC patients [38]. It also linked high CEACAM6 expression to poor OS. To our knowledge, so far, only one study has evaluated the link from CEACAM6 concentration in blood serum to OS and DFS, but it failed to show statistically significant dependency. This is perhaps because of the smaller group of PDAC patients [13]. To our knowledge, our publication is the first report on CEACAM6 blood serum expression as a statistically significant PDAC prognostic biomarker. As CEACAM6 has universal oncogenic features, it has been reported as a relevant prognostic biomarker in other malignancies: cholangiocarcinoma, osteosarcoma, renal cancer, colorectal cancer, gastric cancer [39,40,41,42,43,44,45].

## 5. Conclusions

Our analysis revealed CEACAM6 is the most promising biomarker of all candidates and is predominantly expressed in PDAC tissue. It is also secreted into the blood serum, but its diagnostic potential is low due to increased concentrations in CP blood serum samples. Nevertheless, CEACAM6 blood serum concentrations above the cut-off value significantly correlated with reduced OS in PDAC patients who had undergone radical treatment, while conventional biomarkers (CA19-9, CEA) did not. Since the majority of these patients received adjuvant chemotherapy, CEACAM6 might have a role in distinguishing potential non-responders to adjuvant chemotherapy. This distinguished group could be offered targeted-therapy or/and immunotherapy, since CEACAM6 already demonstrated that it is a good therapeutic target.

## Figures and Tables

**Table 1:** Densitometry readings representing relative intensity of CEACAM6 and β-actin at the Western blot analysis.

	HC				CP				PDAC < 2 cm			PDAC > 3 cm				
Line No.	1	2	3	4	5	6	7	8	9	10	11	12	13	14	15	16
CEACAM6	1.0	0.6	0.5	2.4	11.7	1.8	1.4	1.1	19.9	1.2	6.4	15.6	69.4	110.3	14.9	24.0
β-actin	1.0	1.8	1.9	0.7	1.3	2.3	2.2	1.4	2.8	2.7	2.3	3.8	2.4	2.8	2.1	2.2

**Table 2:** Demographic changes between study groups.

Parameter	PDAC	CP	HC	*p*
No. of patients	142	66	31	
Gender				<0.001
Female No. (%)	71 (50.0)	15 (22.7)	17 (54.8)	
Male No. (%)	71 (50.0)	51 (77.3)	14 (45.2)	
Age (years) Median (IQR)	66.0 (13.8)	49.0 (12.0)	56.0 (11.0)	<0.001
BMI * Median (IQR)	25.4 (5.5)	22.5 (4.4)	27.3 (9.7)	<0.001
Diabetes mellitus				0.009
Present No. (%)	32 (22.5)	27 (40.9)	5 (16.1)	
Absent No. (%)	110 (77.5)	39 (59.1)	26 (83.9)	

* Body mass index.

**Table 3:** Differences in the median blood serum concentrations of analysed biomarkers between the three groups.

Biomarker	PDAC	CP	HC	*p*
CEA * (mkg/L) Median (IQR)	2.9 (3.8)	2.6 (2.7)	1.4 (0.8)	<0.001
CEACAM6 (ng/mL) Median (IQR)	2.1 (2.4)	3.3 (2.3)	1.1 (1.8)	<0.001
CA19-9 ** (kU/L) Median (IQR)	176.0 (1386.9)	7.6 (22.1)	3.6 (5.1)	<0.001

* Carcinoembryonic antigen; ** Carbohydrate antigen 19-9.

**Table 4:** The dependency on oncological and demographical parameters of biomarkers concentrations represented as effect sizes and *p* values.

Parameter	CEA Effect Size (*p*-Value)	CEACAM6 Effect Size (*p*-Value)	Ca19-9 Effect Size (*p*-Value)
Demographical			
Gender	0.01 (0.1) **	−0.01 (0.8) **	0.00 (0.3) **
Age	0.04 (0.46) *	0.09 (0.13) *	−0.05 (0.42) *
Diabetes mellitus	0.01 (0.1) **	0.01 (0.06) **	0.00 (0.9) **
BMI	−0.04 (0.47) *	0.01 (0.85) *	0.04 (0.51) *
Oncological			
T ^1^	0.05 (0.001) **	0.04 (0.008) **	0.05 (0.001) **
N ^2^	−0.01 (0.9) **	0.00 (0.5) **	0.05 (0.002) **
M ^3^	0.07 (0.00005) **	0.02 (0.03) **	0.03 (0.004) **
G ^4^	−0.01 (0.9) **	0.00 (0.6) **	−0.01 (0.7) **
R ^5^	0.00 (0.5) **	0.00 (0.3) **	0.01 (0.1) **
Stage	0.06 (0.002) **	0.00 (0.3) **	0.04 (0.01) **
LNR ^6^	−0.05 (0.59) *	−0.05 (0.56) *	0.13 (0.14) *
Perineural invasion	0.10 (0.000004) **	0.05 (0.0008) **	0.03 (0.02) **
Perivascular invasion	0.09 (0.000007) **	0.06 (0.0005) **	0.02 (0.02) **
Tumour diameter	0.1 (0.1) *	0.1 (0.09) *	0.2 (0.00) *
Tumour localization	0.00 (0.3) **	−0.02 (0.9) **	0.00 (0.4) **
Bilirubin concentration	0.05 (0.4) *	0.01 (0.83) *	−0.04 (0.46) *

* Kendall correlation coefficient; ** eta^2^, based on the H-statistic effect size; ^1^ primary tumour; ^2^ regional lymph nodes; ^3^ distant metastasis; ^4^ differentiation; ^5^ resection status; ^6^ lymph node ratio.

**Table 5:** The characteristics of the subgroups of pancreatic cancer patients.

Parameter	Radical Treatment	Palliative Treatment
No. of patients	73	69
Stage		
IA	4	4
IB	8	0
IIA	11	1
IIB	33	8
III	17	21
IV	0	35
Adjuvant chemotherapy	58	57
Gemcitabine	23	19
Folfirinox	13	8
Gemcitabine and Folfirinox	22	30
Refused/did not tolerate	11	11
Missing data	4	1

**Table 6:** Median survival dependencies of biomarker blood serum concentrations in radical and palliative treatment subgroups.

		Overall Survival (Months)			Disease-Free Survival (Months)		
		Negative *	Positive **	*p*	Negative *	Positive **	*p*
Radical treatment	CEA median (IQR)	15.6 (22.8)	15.4 (16.6)	0.3	8.7 (17.3)	7.9 (11.0)	0.38
	CEACAM6 median (IQR)	17.0 (21.3)	12.6 (14.1)	0.017	8.7 (14.4)	7.0 (19.0)	0.094
	CA19-9 median (IQR)	16.4 (18.8)	13.3 (21.8)	0.64	8.1 (16.4)	9.2 (11.9)	0.23
Palliative treatment	CEA median (IQR)	11.7 (13.6)	13.6 (9.9)	0.71			
	CEACAM6 median (IQR)	11.8 (7.9)	13.5 (12.6)	0.87			
	CA19-9 median (IQR)	13.6 (16.4)	12.0 (10.5)	0.12			

* Biomarker concentrations below cut-off value; ** biomarker concentrations above the cut-off value.

## Data Availability

In-depth proteomics analysis results are available as Supplementary Table S1. Raw mass spectrometry data could be available upon request to the corresponding author. Patients clinical and demographical data presented in this study could be available on request from the corresponding author. The data are not publicly available due to privacy issues.

## Supplementary Materials

The following is available online at https://www.mdpi.com/article/10.3390/life11060542/s1; Table S1: Proteins identified in HDMS analysis; Table S2: Densitometry readings representing relative intensity of CEACAM6 and β-actin at the full view Western blot analysis; Figure S1: Full view of Western blot analysis of CEACAM6 expression in HC, CP, and PDAC tissue lysates; Figure S2: Full view of Western blot analysis of β-actin used as a loading control for HC, CP, and PDAC tissue lysates.
