# Supplementary figures and images for: CEACAM6’s Role as a Chemoresistance and Prognostic Biomarker for Pancreatic Cancer: A Comparison of CEACAM6’s Diagnostic and Prognostic Capabilities with Those of CA19-9 and CEA
Source: Life (Basel). 2021 Jun 9;11(6):542. doi: 10.3390/life11060542 (PMC8226832; doi:10.3390/life11060542)

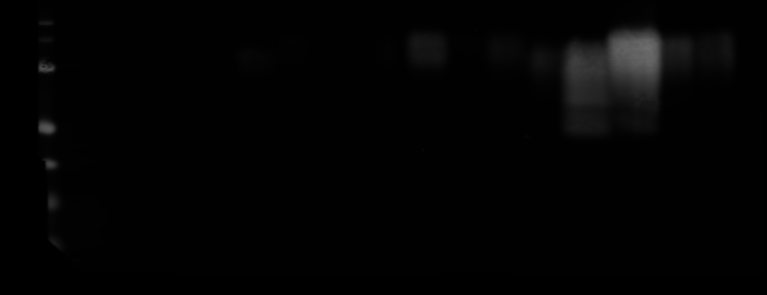

Supplement: Supplementary file 1 [file life-11-00542-s001.zip › Supplementary Files/Supplementary Materials_Figure_S1.TIF]

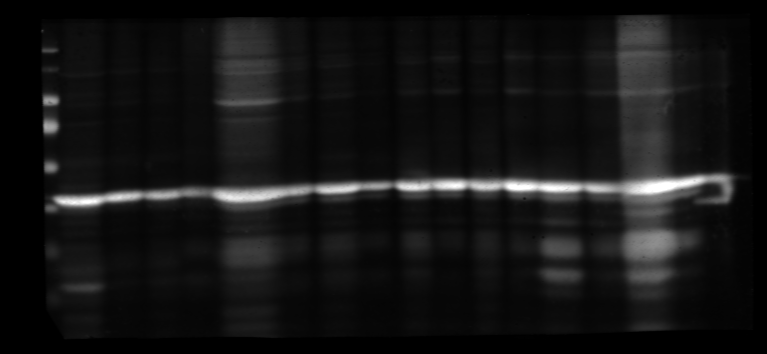

Supplement: Supplementary file 1 [file life-11-00542-s001.zip › Supplementary Files/Supplementary Materials_Figure_S2.TIF]
